# Applications of the reflective practice questionnaire in medical education

**DOI:** 10.1186/s12909-019-1481-6

**Published:** 2019-02-07

**Authors:** Shane L. Rogers, Lynn E. Priddis, Nicole Michels, Michael Tieman, Lon J. Van Winkle

**Affiliations:** 1 Edith Cowan University, Joondalup, WA Australia; 2 Department of Medical Humanities, Rocky Vista University, 8401 S. Chambers Road, Parker, CO 80134 USA

**Keywords:** Reflective capacity, Medical education, Job satisfaction, Anxiety, Stress, Communication, Over-confidence

## Abstract

**Background:**

We sought to determine whether the Reflective Practice Questionnaire (RPQ) is a reliable measure of reflective capacity and related characteristics in medical students. We also planned to learn how the RPQ could be used in medical education.

**Methods:**

The RPQ is a 40 item self-report questionnaire that includes a multi-faceted approach to measuring reflective capacity. It also includes sub-scales on several other theoretically relevant constructs such as desire for improvement, confidence, stress, and job satisfaction. The reliabilities of reflective capacity and other sub-scales were determined by calculating their Cronbach alpha reliability values. In the present study, the RPQ was answered by 98 graduating fourth-year medical students from an American University, and these RPQ scores were compared with general public and mental health practitioner samples from a prior study using ANOVA and Bonferroni adjusted comparisons.

**Results:**

Medical students reported a higher reflective capacity than the general public sample, but students were statistically indistinguishable from the mental health practitioner sample. For medical students, reflective capacity was associated with features of confidence, stress, and desire for improvement. Job satisfaction was positively associated with confidence in communication with patients, and negatively associated with stress when interacting with patients. A cluster analysis revealed that around 19% of the medical students exhibited a relatively high level of anxiety interacting with patients, 23% were less engaged, 5% were dissatisfied, and 7% expressed a level of over-confidence in their knowledge and skills that was concerning.

**Conclusions:**

The RPQ is a reliable measure of reflective capacity (Chronbach’s alpha value = 0.84) and related characteristics (Cronbach’s alpha values from 0.75 to 0.83) in medical students. The RPQ can be used as part of pre-post evaluations of medical education initiatives, to complement student self-reflection activities in the curriculum, and to identify students who might benefit from targeted intervention.

**Electronic supplementary material:**

The online version of this article (10.1186/s12909-019-1481-6) contains supplementary material, which is available to authorized users.

## Background

Over the past few decades, developing reflective capacity in students has become a core element of many university courses in the health sciences broadly [1–7], and specifically within medical education [8–17]. Reflective capacity refers to the ability, desire, and tendency of students to engage in reflective thought during their academic studies and clinical practices. In reflective thought, individuals critically appraise assumptions and beliefs (their own, their colleagues, and their patients) and take an open-minded stance to general problem-solving and interactions with patients. The present study reports medical student responses to the newly developed reflective practice questionnaire (RPQ) [1].

The RPQ assesses self-reported reflective capacity and can be used to compare across every profession where interactions with clients occur [1]. The RPQ measures multiple aspects of reflective capacity, and additional relevant dimensions such as confidence, uncertainty, stress, desire for improvement, and job satisfaction.

### Measures of reflection in medical education

Several self-report questionnaires have been developed to assess reflective thought in medical students and practitioners, with some different areas of focus among the existing instruments. Some scholars focused on the extent of reflection during learning [18]. For example, Kember et al. [19] designed the *questionnaire for reflective thinking* by asking university students to rate agreement on statements that referred to their university experience. The questionnaire asks respondents about their level of reflection on their own thoughts, actions, habits, understanding, and growth as individuals in their university courses. In a similar fashion, Sobral [20] constructed the *reflection-in-learning* questionnaire to assess self-reported reflection upon learning in a *medical* program. Sobral’s questionnaire differs from Kember et al.’s approach by focusing more on self-reported extent of reflection upon the meaning and purpose of the to-be-learned content, and upon study habits.

Mamede and colleagues designed a questionnaire focused primarily on reflection during *medical* diagnostic reasoning [21–24]. Their questionnaire covers different aspects of reasoning such as an openness towards and tendency to generate multiple alternative hypotheses, willingness to reflect on different hypotheses and question one’s assumptions, and willingness to test one’s hypotheses [24]. Mamede and colleagues’ approach sets itself apart from other approaches by specifically focusing upon reflection during decision making.

Aukes and colleagues developed the *Groningen Reflection Ability Scale* (GRAS) to evaluate personal reflection in *medicine* [25]. Aukes et al. describe the GRAS as a unidimensional scale yet also discuss three broad sub-types of questions within the questionnaire – self-reflection, empathetic reflection, and reflective communication [25]. The self-reflection component of this questionnaire contains items that ask about awareness of one’s own thinking and behaviour (e.g., “I am aware of the emotions that influence my thinking”). The empathetic reflection component contains items about empathizing with others (e.g., “I can empathize with someone else’s situation”), although it contains items that do not seem to have high face validity for such a component (e.g., “I am aware of my own limitations”). The reflective communication component contains items about being responsible and accountable for one’s own communication (e.g., “I am accountable for what I say”), and also stubbornness (e.g., “I do not like to have my standpoints discussed”). Both the initial study [25], and a more recent evaluation [26], do not support separation of these components of the questionnaire on statistical grounds. Hence, the recommendation to use it as a unidimensional measure of personal reflection. Of the self-report questionnaires mentioned here, the GRAS has the most in common with the reflective practice questionnaire (RPQ) we used in the present study. However, as we will describe below, the RPQ arguably has greater clarity regarding what it measures.

Here we concentrated on self-report measures of reflection since the present study used a self-report questionnaire. However, we acknowledge that other approaches to assessment of reflective capacity have been used in the literature. A popular technique is to qualitatively rate the content of writing on aspects of reflection to assess a person’s level of reflective capacity [8–11, 27–37]. A wide array of criteria have been applied across studies using this technique and a complete summary is beyond the scope of the present discussion. Other researchers have taken a more structured approach by rating written responses to short written vignettes [38], videos [39], or semi-structured exercises [40, 41].

### The reflective practice questionnaire (RPQ)

Beyond the use of self-report measures of reflection in *medical* education, there is a need to foster reflective practice in all healthcare professionals and their students including those in public health, health services management, health administration, dentistry, medicine, nursing, pharmacy, and other disciplines [42]. In response to this shortcoming, and a need to measure reflective practice in all professionals who serve the public, Priddis and Rogers [1] created the RPQ to assess a wider array of psychological constructs relevant to reflective practice.

The RPQ was developed in response to this call for more comprehensive reflective practice self-report measures. The RPQ sets itself apart from other surveys in several ways. First, it was designed mindfully to use with different professions and gain/yield new insights from comparisons across contexts. Second, the RPQ items focus mostly upon *interactions with clients*. It is this common reference point that affords the questionnaire to have relevance across different professions. This focus also helps to set the RPQ apart from other self-report measures in the literature. The RPQ contains a 16-item reflective capacity (RC) sub-scale and six other 4-item sub-scales.

The 16-item RC sub-scale has four, 4-item sub-components that together provide a self-reported measure of a respondent’s *reflective capacity*. Priddis and Rogers [1] labeled these four sub-components reflection-in-action, reflection-on-action, reflection with others, and self-appraisal. The reflection-in-action and reflection-on-action sub-scales were inspired by the concepts of the same name in the seminal works of Schon [43, 44]. The primary distinction between the two types is that reflection-in-action refers to reflection occurring in the moment whereas reflection-on-action refers to reflection on past events. Hence in the RPQ reflection-in-action items begin “During interactions with clients…” and reflection-on-action items begin “After interaction with clients…”.

While the in-action and on-action distinctions were inspired by Schon, this is where the RPQ affiliation with any specific theorist ends. The RPQ items were designed to be broad and open to interpretation to enable wide application of the survey across different professions, and among people with varying perspectives on reflection. For example, the RPQ reflection-in-action item “During interactions with clients I consider how my personal thoughts and feelings are influencing the interaction” asks the participant to rate their extent of *consideration* but is not prescriptive regarding what the consideration entails. More specifically, the survey does not force upon the respondent any notions regarding how conscious (deliberate) or non-conscious (intuitive), rational or emotional, systematic or unsystematic, their reflective process should be.

This open stance is consistent with early theorists such as Schon, Dewey, and Wertheimer who were largely descriptive (rather than prescriptive) and very broad regarding what constitutes an act of reflection (for a discussion, see [45]). We recognize that over the years there have been numerous attempts to be more prescriptive regarding what constitutes an ideal reflective process (for some examples, see [46–52]). For scholars wishing to use the RPQ to assess extent of reflection from a specific viewpoint one need only introduce an introductory preamble to the survey to influence respondent interpretation of the items. Like Priddis and Rogers [1], we did not place any perspective on respondents in the present study and allowed them to answer the items interpreting what constitutes reflection as they wished.

The reflection with others sub-component within the RPQ is included in response to the frequent recognition in the literature that reflecting with other people can facilitate insights and understanding [53–56]. This can occur as fragmented understanding becomes solidified as a co-creation during dialogue, as people form a *shared reality* [57, 58]. Additionally, already solidified understandings can be modified by feedback from others that enables reconceptualization and helps one to consider ideas from different points of view [59–62]. The RPQ refers to reflection with “others” to be inclusive of reflection that might occur with peers [63–66], or in formal supervision [3, 67–69]. For a researcher interested in investigating differential benefits of reflection with different types of ‘others’, one need only repeat the sub-component multiple times substituting the word ‘others’ with a specific type of person (e.g., work colleagues, supervisor, friends). Like Priddis and Rogers [1], we left this open in the present study so that participants would interpret as best suited them.

Inclusion of the RPQ self-appraisal sub-component acknowledges that a tendency for personal reflection and reflection with others likely fosters an increased tendency to reflect upon and question one’s own capabilities for practice [70–72]. This is consistent with theorists that promote a cycle of learning such as Kolb [73], Gibbs [74], and Argyris and Schon [75] who emphasize the role of reflection for assessing one’s own strengths, weaknesses, and approaches, thus stimulating self-growth [17, 76]. Priddis and Rogers [1] reported strong positive correlations among the reflection-in-action, reflection-on-action, reflection with others, and self-appraisal sub-components. Hence we were expecting to observe the same pattern of results in our medical student sample. We then proceeded a step further to conduct a factor analysis on a combination of these sub-components to assess whether averaging across all items in a single RC sub-scale is appropriate.

In addition to four dimensions of reflection, the RPQ incorporates several other attributes relevant to reflective practice – general confidence, desire for improvement, uncertainty, and job satisfaction. Uncertainty is proposed to have an intimate link with reflection. For example, Clara [45] states that “Reflection consists of giving coherence to a situation that is initially incoherent and unclear” (p. 262). Uncertainty stimulates reflection as one reflects to try and resolve the uncertainty. It is therefore not surprising that Priddis and Rogers [1] reported positive associations between the RC sub-components and the uncertainty sub-scale of the RPQ. On the other hand, it has been postulated that building practitioner reflective capacity helps to build practitioner desire for continual improvement [77] and confidence [78, 79]. Increased confidence in one’s work has been shown to be positively related to job satisfaction [80]. Additionally, others have noted that reflective capacity might help practitioners to be more emotionally resilient, and resistant to burnout, with related higher job satisfaction [81–83].

Also included in the RPQ are *specific* sub-scales for confidence and stress that target *communication* with clients in general and patients in particular. Doctor-patient communication is widely recognized as a fundamental aspect of medical practice [84–89]. A lack of patient understanding due to unclear communication can reduce the level of trust between doctor and patient, lower adherence to treatment plans, raise patient uncertainty and anxiety levels, and thus have a negative impact on patient outcomes [84].

The reason for including a wide array of sub-scales within the RPQ was to provide a succinct self-report instrument of use to scholars wishing to investigate current issues regarding reflective practice [1]. Building reflective capacity is believed to lead practitioners to embrace uncertainty and build confidence. Yet a focus upon uncertainty may instead have a darker side whereby confidence is undermined, and rumination provides a fertile ground for anxiety [90–92]. This tension between reflection as a force for good, versus bad, has historically been over-looked within the reflective practice literature.

Additionally, a seemingly paradoxical notion is that if reflection promotes self-growth and confidence, which should reduce uncertainty, then reflection might act to reduce the need for itself over time. A basic tenant of psychological theories of skill acquisition is to emphasize how skills shift from a conscious to unconscious mode of thought as expertise develops [93]. How reflection can be maintained as a force for good (e.g., building confidence and resilience) while maintaining a continual desire for improvement remains an area for future research. The RPQ is also designed to be used in a piecemeal fashion by scholars depending on their specific interests [1]. For example, scholars interested solely in reflection may wish to use only the RC sub-scale of the RPQ.

### The present study

Priddis and Rogers studied samples of the general public and mental health practitioners using the RPQ [1]. The present study explores the utility of this new questionnaire with medical students. These students were about to graduate from an American medical school that states its mission to “inspire students to serve with compassion, integrity, and excellence” (http://www.rvu.edu/about/mission-and-vision/). Hence, we expected the self-reported reflective capacity of students, who attempted to live up to this mission, to be higher than the general public sample from Priddis and Rogers [1]. We were uncertain how the medical students would compare with mental health practitioners as all individuals in the latter sample reported reflective capacity building as part of their professional training [1] whereas the sample of medical students did not have formal reflective capacity building. The mental health practitioner sample reported in this study includes additional responses to increase the sample size from 45 as reported in Priddis and Rogers [1] to 100 mental health practitioners in the current study.

Our findings with medical students show how the RPQ can be used by medical educators to assess the success of reflective practice education efforts and identify individuals/groups that may benefit from targeted educational intervention. For example, using a cluster analysis, we show that the RPQ can identify students who lack confidence, experience stress when interacting with patients, or avoid reflection.

## Methods

### Participants

One hundred fifty medical students from Rocky Vista University, Colorado, US were invited to complete the RPQ about two weeks before graduation and 100 students responded (67% response rate). Two of the responses were incomplete, leaving 98 anonymous responses for the present analysis. The ages and genders of the responding students (average age 28.3 years and 47% female) were similar to the entire class (average age 28.7 years and 44% female). One student’s responses constituted a multivariate outlier and were not included in the analysis. Prior to conducting this research, the Rocky Vista University Institutional Review Board approved the study (exempt category).

For comparison purposes, anonymous responses from 45 Australian mental health practitioners and 188 members of the Australian general public [1] were reassessed in the present study. Prior to this assessment, 55 new responses were added to the mental health practitioner sample. Members of the general public had been recruited using the survey company, Qualtrics. Mental health professionals were recruited by emailing contacts of one of us (LEP) at a range of organizations.

### Measures

Participants completed the RPQ shown in section 1 of the online supplement document (Additional file 1). The RPQ was designed for flexible use across different professions by replacing the term ‘clients’ in the original survey with the normative term to describe ‘clients’ of the service [1]. In the present study, we used the term ‘patients’. This 40-item self-report instrument provides measures for reflective capacity via the sub-components reflection-in-action, reflection-on-action, reflection with others, and self-appraisal (described in detail under Background). To improve RPQ reliability as a measure of reflection for the relatively homogeneous sample of medical students, we combined the four sub-components of reflection into a single 16-item subscale termed ‘reflective capacity’ (RC). Together, the four reflection sub-components form an overall measure of RC.

Additional sub-scales form parts of the RPQ that aim to assess a range of other theoretically relevant constructs. A desire for improvement sub-scale gauges one’s inclination to further one’s expertise. Confidence in one’s general ability is measured via a confidence (general) sub-scale, and confidence more specifically related to communication is assessed via a confidence (communication) sub-scale. Uncertainty and stress interacting with patients sub-scales are also included. Finally, the RPQ contains a general job satisfaction sub-scale. All of these sub-scales were found by Priddis and Rogers to correlate with RC [1]. The RPQ uses the 6-point response scale; (1) Not at all, (2) Slightly, (3) Somewhat, (4) Moderately, (5) Very much, (6) Extremely. See online supplement document section 1 associated with this article for a full copy of the RPQ with scoring instructions.

### Statistical methods

Factor analysis of the RC subscale was conducted using the principal factor method in the statistical program, Stata [94]. Using this method, the factor loadings are computed using the squared multiple correlations as estimates of the communality. Since we obtained a single factor solution, no rotation was applied.

We determined descriptive statistics for the RPQ sub-scales. For comparison purposes, we also included data previously reported by Priddis and Rogers for general public and mental health practitioner samples [1], and we conducted a series of one-way ANOVAs with follow-up Bonferroni adjusted comparisons to compare the RPQ sub-scales across samples. The ANOVA results are presented in online supplement section 3. For the sake of brevity, we simply report, under results, the main differences that were found. To indicate the importance of differences among sample means, Effect Size values were calculated as Cohen’s *d* (*d* = difference between means/pooled standard deviations of the means) [94]. Values of *d* of about 0.2, 0.5, and 0.8 have negligible, moderate, and crucial practical importance, respectively.

Focusing solely on the medical students, we also examined the inter-relations between RPQ sub-scales and conducted an exploratory cluster analysis to uncover meaningful sub-groups. We used the RPQ scores in a hierarchical agglomerative cluster analysis using weighted average linkage method and using absolute-value distance as the dissimilarity measure. This analysis was conducted using the statistical program, Stata,^1^ to explore whether meaningful patterns emerged [94].

## Results

Factor analysis of the medical student, mental health practitioner, and general public RC sub-scales revealed single-factor solutions (Table 1). We retained all RC items for comparison purposes because our conclusions were the same regardless of whether the two items with factor loadings below 0.3 were included for medical students. While one of the latter items involves questioning one’s pre-existing beliefs, the other does not. Moreover, another item, concerning the impact of one’s personal thoughts and feelings, loaded well. For these reasons, we suggest items 15 and 16 in Table 1 both loaded poorly because of similarities in their wording rather than an inability of medical students to reflect on their own beliefs.

**Table 1:** Single Factor Solution factor loadings for the reflective capacity subscale of the reflective practice questionnaire with medical students, mental health practitioners, and the general public ^a^

Items	Medical Students (*n* = 97)	Mental Health Practitioners (*n* = 100)	General Public (*n* = 188)
ᅟ1. After interacting with patients/clients I think about how things went during the interaction. (33)	0.73	0.76	0.84
ᅟ2. When reflecting with others about my work I develop new perspectives. (12)	0.72	0.68	0.71
ᅟ3. I gain new insights when reflecting with others about my work. (38)	0.68	0.69	0.75
ᅟ4. After interacting with patients/clients I wonder about the patient’s experience of the interaction. (16)	0.67	0.79	0.76
ᅟ5. I find that reflecting with others about my work helps me to work out problems I might be having. (29)	0.59	0.62	0.75
ᅟ6. After interacting with patients/clients I spend time thinking about what was said and done. (3)	0.57	0.53	0.69
ᅟ7. I think about my weaknesses for working with patients/clients. (13)	0.55	0.63	0.84
ᅟ8. After interacting with patients/clients I wonder about my own experience of the interaction. (24)	0.53	0.80	0.77
ᅟ9. During interactions with patients/clients I consider how their personal thoughts and feelings are influencing the interaction. (35)	0.53	0.77	0.79
ᅟ10. I think about how I might improve my ability to work with patients/clients. (23)	0.52	0.74	0.80
ᅟ11. During interactions with patients/clients I consider how my personal thoughts and feelings are influencing the interaction. (14)	0.46	0.76	0.81
ᅟ12. I critically evaluate the strategies and techniques I use in my work with patients/clients. (36)	0.36	0.55	0.59
ᅟ13. I think about my strengths for working with patients/clients. (7)	0.33	0.50	0.71
ᅟ14. When reflecting with others about my work I become aware of things I had not previously considered. (1)	0.33	0.61	0.61
ᅟ15. During interactions with patients/clients I recognize when my pre-existing beliefs are influencing the interaction. (9)	0.24	0.56	0.60
ᅟ16. During interactions with patients/clients I recognize when my patient’s/client’s pre-existing beliefs are influencing the interaction. (26)	0.23	0.57	0.74
Factor loadings median	0.53	0.66	0.75
Factor loadings range	0.23–0.73	0.50–0.80	0.59–0.84
Factor Eigenvalue	4.46	7.12	8.71

^a^ Students were from the Rocky Vista University College of Osteopathic Medicine, Colorado, United States; Mental health practitioners, and members of the general public were from a variety of locations around Australia. Question numbers on the RPQ are in ()

The RC sub-scale (total of 16 items) showed good internal consistency for the medical student sample (Cronbach’s alpha reliability value = 0.84 with two items having low correlations included, 0.85 without them). The mean medical student RC value was significantly higher than the general public value (*d* = 0.74, *p* < .001) but not higher than the mental health practitioner sample (Table 2).

**Table 2:** Mean RPQ scores for the medical students of the present study, compared with the mental health practitioner and general public samples

RPQ sub-scale	Medical students (U.S.)	Mental health practitioners (Aus.)	General public (Aus.)
RC	4.16^2^(0.53) [.84]	4.27 (0.68) [.92]	3.51 (1.02) [.96]
DfI	4.91^1,2^ (0.83) [.81]	4.38 (0.91) [.84]	3.32 (1.27) [.91]
CG	3.28^2^(1.06) [.83]	3.27 (0.90) [.76]	4.07 (1.02) [.82]
CC	4.58 (0.61) [.75]	4.53 (0.55) [.64]	4.44 (0.92) [.82]
Unc	3.47^1,2^ (0.81) [.81]	2.91 (0.79) [.74]	2.52 (1.05) [.86]
SiP	3.42^1^(0.94) [.81]	2.97 (0.91) [.82]	3.17 (1.24) [.86]
JS	4.81^2^(0.73) [.78]	4.89 (0.71) [.72]	4.00 (1.27) [.86]

^1^Significantly different (*p* < .05) compared to the mental health practitioners

^2^Significantly different (*P* < .05) compared to the general public sample

Sub-scales: *RC* Reflective capacity, *DfI* Desire for improvement, *CG* Confidence – general, *CC* Confidence – communication, *Unc* Uncertainty, *SiP* Stress interacting with patients, *JS* Job satisfaction

Standard deviations are provided in () brackets. Cronbach’s alpha reliability values are provided in [] brackets

The mean scores for all three samples on each of the other RPQ sub-scale are also presented in Table 2. The Cronbach’s alpha reliability values for other sub-scales of the medical student sample were acceptable to good (i.e., 0.75 to 0.83) (Table 2). The inter-correlations among items for RPQ sub-scales with four items are provided in online supplement document section 2. Overall, medical students reported a strong desire for improvement (mean sub-scale score near 5 or ‘very much’), that was greater than the other two samples (*d* = 0.61 and 1.48). They were also significantly higher compared to both other samples on the uncertainty sub-scale (*d* = 0.65 and 1.01). They reported similar levels of general confidence and communication confidence as the mental health practitioner sample but significantly less general confidence than the general public sample (*d* = 0.78). Their stress interacting with patients was significantly higher than the mental health practitioner sample (*d* = 0.49). Their job satisfaction was on par with the mental health practitioner sample, and significantly higher than the general public sample (*d* = 0.71).

The inter-correlations among the RPQ sub-scales are presented for the medical student sample in Table 3. Like results reported by Priddis and Rogers [1], the RC sub-scale was positively correlated with uncertainty and stress (*r* = 0.46 and 0.41). Also, RC was positively correlated with desire for improvement (*r* = 0.43). Uncertainty and Stress were positively associated with desire for improvement (*r* = 0.41 and 0.25, respectively), and negatively associated with the two confidence sub-scales (*r* = − 0.22 to − 0.33). Communication confidence was, however, positively associated with RC (*r* = 0.25). This is important to note because communication confidence has the strongest association with job satisfaction (*r* = 0.46), as was the case for the public sample from Priddis and Rogers [1]. However, unlike results reported by Priddis and Rogers, we also found a moderate negative association between job satisfaction and stress interacting with patients (*r* = − 0.42).

**Table 3:** Pearson correlations among the RPQ sub-scales for the medical student sample

	RC	DfI	CG	CC	Unc	SiP	JS
RC	1						
DfI	.43*	1					
CG	−.09	−.17	1				
CC	.25*	.10	.31*	1			
Unc	.46*	.41*	−.33*	−.22*	1		
SiP	.41*	.25*	−.26*	−.30*	.64*	1	
JS	.17	.22*	.14	.46*	−.15	−.42*	1

*p < .05

Sub-scales: *RC* Reflective capacity; *DfI* Desire for improvement; *CG* SConfidence – general, *CC* Confidence – communication, *Unc* Uncertainty, *SiP* Stress interacting with patients, *JS* Job satisfaction

**Fig. 1:**
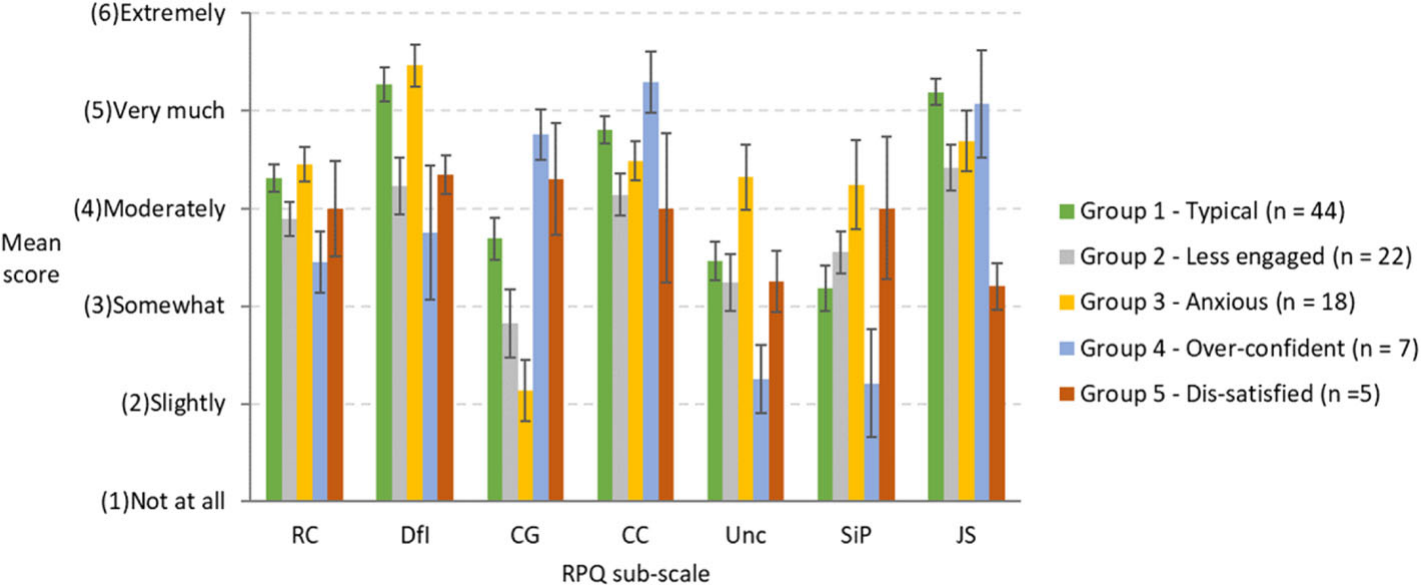
Mean RPQ sub-scale scores for each group identified via cluster analysis. Error bars represent 95% confidence limits. RC = Reflective capacity; DfI = Desire for improvement; CG = Confidence – general; CC = Confidence – communication; Unc = Uncertainty; SiP = Stress interacting with patients; JS = Job satisfaction

To explore individual differences within our sample of medical students we conducted a cluster analysis using the RPQ sub-scale scores. Visual inspection of the dendrogram associated with the cluster analysis revealed five groups as the best classification system for the data. The dendrogram is provided in online supplement document section 4. To assist interpretation of these groups, we provide the mean values for each RPQ sub-scale in Fig. 1.

The largest number of participants (45%) were part of a *typical* group, with scores on the RPQ that resembled the overall statistics reported in Table 2. Of main interest were the four smaller sub-groups and how they compared with the typical student. One sub-group (23%) we refer to as *less engaged*. They expressed lower levels of desire for improvement, job satisfaction, and confidence than the typical group. A related sub-group (5%) seemed *dissatisfied*, as they had much lower job satisfaction than all other sub-groups. They also reported lower confidence in communication than typical students. A third sub-group (19%) we refer to as *anxious*, because they reported much higher uncertainty and stress interacting with patients than the typical students. The final group (7%) we describe as *over-confident*, since they reported high confidence in their knowledge, skills, and ability to communicate with patients. They also expressed low uncertainty, stress, desire for improvement, and RC. For statistical analysis of the differences between cluster groups see online supplement section 5.

## Discussion

This is the first study of medical students using the newly-developed RPQ [1]. We compared the mean responses on the RPQ sub-scales from our medical students with a previous general public sample of Priddis and Rogers and an expanded sample of mental health practitioners [1]. Since our medical student sample came from a University that prioritizes compassion and personal excellence, we anticipated their self-reported RC would be relatively high. The medical students rated themselves higher in RC than the general public sample but not higher than the mental health professional sample. Members of the latter sample reported formal reflective training and practice [1] whereas medical students had no such formal training.

As for findings of Priddis and Rogers [1], positive associations were found between the RC sub-scale and the uncertainty and stress sub-scales in medical students. Causal implications of this association are unclear. It could be that uncertainty and stress encourage greater reflection, or that greater reflection leaves one at risk of rumination that increases uncertainty and stress. Likely both processes work in concert to some degree and teasing them apart with longitudinal investigations is an intriguing avenue for future research. For example, since uncertainty and stress are both negatively associated with general confidence, perhaps self-efficacy plays a role in these relationships [20]. While associated with stress, RC is also associated with desire for improvement. Rather than warning against reflection, we simply seek to raise awareness of potential risks for increased stress. We recommend that medical educators be mindful of this association and take steps to make their students aware of it.

The right amount of stress stimulates growth and development as professionals, while not enough or too much stress is counterproductive [95]. Excessive stress is not only of concern for general mental health, but also for the role it plays in diminishing a positive outlook on one’s work [96–98]. For example, in the present study we found a negative relationship between stress and job satisfaction sub-scales. In the cases of some students, however (e.g., the over-confident ones in the present study), their very low uncertainty and stress and high job satisfaction may not have been optimal. There may be negative consequences associated with the latter combination such as increased chances of risky decision making [99–102], or an arrogant demeanor offending colleagues and patients [103–105].

In addition to the correlation between RC and desire for improvement, RC correlated positively with confidence in communication. The confidence in communication sub-scale in turn was found to have a positive association with the job satisfaction sub-scale, and job satisfaction correlated positively with desire for improvement. These correlations may simply show associations without causal relationships. Alternatively, RC may help a practitioner feel more confident in their interactions with patients which can help foster greater enjoyment of their medical practice. The two sub-scales to associate relatively strongly with job satisfaction were the confidence in communication sub-scale (positive association) and the stress interacting with patients sub-scale (negative association). While much of the literature in medical education acknowledges the benefit of good doctor-patient communication for the patient [84–88], our results also highlight how doctor-patient communication may contribute to the welfare of the doctor. We suggest that medical educators might foster better engagement with lessons on patient communication by stressing the potential benefits for the students themselves (i.e., less stress and more confidence and job satisfaction), in addition to the well-being of their patients.

As part of our analysis of results, we also conducted an exploratory cluster analysis to gain a more nuanced understanding of the cohort of participants. We were inspired by Speelman and McGann who discussed how an over-reliance on mean scores can obscure interesting individual differences present in data [106]. We acknowledge that our cluster analysis is based on a small specific sample of medical students and that there is limited generalizability. However, our purpose was simply to try to better understand the individual differences within our sample, with potential to provide insights which might lead to further research.

Indeed, the cluster analysis revealed some interesting findings. Based on our sample, around 19% of the students fell into a category we refer to as *anxious*. Compared to typical students (Fig. 1), this sub-group expressed substantially higher uncertainty and stress interacting with patients. In contrast, around 5% of the sample reported much lower job satisfaction and, thus, appeared to be *dissatisfied*. A related group of about 23% of students were *less engaged*. They expressed less desire for improvement, low job satisfaction, and less confidence than the more typical group of students. Finally, a sub-group of around 7% of the sample seemed *over-confident*. This sub-group showed a very high level of confidence in their knowledge, skills, and ability to communicate with patients, and low levels of stress, uncertainty, desire for improvement, and RC. Thus, one use for the RPQ in medical education could be to assist in identifying students who might report concerning levels of anxiety, over-confidence, or dissatisfaction/disengagement with medical practice. This information could help educators make targeted interventions to better address the needs of individual students [107–110]. Educators must, of course, be careful not to label individual students [111]. RPQ sub-scales are not intended to measure fixed personality characteristics but instead *represent malleable capacities*.

### Limitations

We studied a single sample of medical students so our results and interpretations should be applied cautiously to other such students. In addition, we compared *American* medical students to *Australian* general public and mental health practitioner samples. As research accumulates with the RPQ, more comparisons within and across professions in different countries should help to drive forward understanding of how different disciplines approach education for reflective practice. Another limitation is the *self-report* nature of the measure, and we recommend that the RPQ be used in conjunction with other reflection activities and forms of peer and teacher feedback on RC. Finally, some of our data (i.e., Table 3) show associations that may or may not reflect causal relationships. For example, perhaps medical students who are satisfied with their work (‘job satisfaction’) are more likely to want to improve to do better for their patients (‘desire for improvement’), or perhaps these are simply associated, without a causal relationship.

## Conclusions

The RPQ can be used as a self-report instrument in medical education. It applies to program evaluations, particularly those that aim to improve reflective functioning of practitioners. In addition, the RPQ can be utilized for education where students and practitioners complete the questionnaire as part of their own self-reflection and professional development. Finally, it can be used to identify students who might benefit from targeted intervention to address issues such as anxiety, over-confidence, or dissatisfaction/disengagement with medical practice.

For a detailed description of cluster analysis options using Stata please see: https://www.stata.com/manuals13/mvcluster.pdf

## Additional file

Additional file 1: Online supplement document for article: *Applications of the reflective practice questionnaire in medical education.* This document contains five sections: Section 1. The RPQ as used in the present study, pages 2 – 3. Section 2. Inter-correlations among items for RPQ sub-scales with four items, page 4. Section 3. Comparison across RPQ sub-scales for different groups, page 5. Section 4. Cluster analysis dendrogram, page 6. Section 5. Statistical comparison of the cluster analysis groups, page 7. (DOCX 101 kb)

## Abbreviations

CC: Confidence - communication
CG: Confidence – general
DfI: Desire for improvement
JS: Job satisfaction
RC: Reflective capacity
RPQ: Reflective practice questionnaire
SiC: Stress interacting with patients
Unc: Uncertainty
