# Supplementary material for: Applications of the reflective practice questionnaire in medical education
Source: BMC Med Educ. 2019 Feb 7;19:47. doi: 10.1186/s12909-019-1481-6 (PMC6367754; doi:10.1186/s12909-019-1481-6)
Supplement: Supplementary file 1 — Online supplement document for article: Applications of the reflective practice questionnaire in medical education. This document contains five sections: Section 1. The RPQ as used in the present study, pages 2 – 3. Section 2. Inter-correlations among items for RPQ sub-scales with four items, page 4. Section 3. Comparison across RPQ sub-scales for different groups, page 5. Section 4. Cluster analysis dendrogram, page 6. Section 5. Statistical comparison of the cluster analysis groups, page 7. (DOCX 101 kb) [file 12909_2019_1481_MOESM1_ESM.docx]

Online supplement document for article:

*Applications of the reflective practice questionnaire in medical education*

This document contains four sections:

Section 1. The RPQ as used in the present study, pages 2 – 3.

Section 2. Inter-correlations among items for RPQ sub-scales with four items, page 4.

Section 3. Comparison across RPQ sub-scales for different groups, page 5.

Section 4. Cluster analysis dendrogram, page 6.

Section 5. Statistical comparison of the cluster analysis groups, page 7.

**Section 1. The RPQ as used in the present study.**

Below the items for the RPQ sub-scales are provided under headings denoting the label for each sub-scale. The numbers next to each question item refer to the order in the questionnaire each item appeared for participants. The response scale for items is scored on a scale of 1-6 where: (1) Not at all, (2) Slightly, (3) Somewhat, (4) Moderately, (5) Very much, (6) Extremely. For each sub-scale the four items are averaged to provide the sub-scale score. Please note that for the ‘job satisfaction’ sub-scale one of the items requires reverse scoring prior to averaging.

*Reflection-in-action (RiA)*

- 9. During interactions with patients I recognize when my pre-existing beliefs are influencing the interaction.
- 14. During interactions with patients I consider how my personal thoughts and feelings are influencing the interaction.
- 26. During interactions with patients I recognize when my patient’s pre-existing beliefs are influencing the interaction.
- 35. During interactions with patients I consider how their personal thoughts and feelings are influencing the interaction.

*Reflection-on-action (RoA)*

- 3. After interacting with patients I spend time thinking about what was said and done.
- 16. After interacting with patients I wonder about the patient’s experience of the interaction.
- 24. After interacting with patients I wonder about my own experience of the interaction.
- 33. After interacting with patients I think about how things went during the interaction.

*Reflection with others (RO)*

- 1. When reflecting with others about my work I become aware of things I had not previously considered.
- 12. When reflecting with others about my work I develop new perspectives.
- 29. I find that reflecting with others about my work helps me to work out problems I might be having.
- 38. I gain new insights when reflecting with others about my work.

*Self-appraisal (SA)*

- 7. I think about my strengths for working with patients.
- 13. I think about my weaknesses for working with patients.
- 23. I think about how I might improve my ability to work with patients.
- 36. I critically evaluate the strategies and techniques I use in my work with patients.

*Desire for improvement (DfI)*

- 5. I think I still have a lot of things to learn in order to improve my ability to work with patients.
- 19. I would like to learn new skills in order to improve my ability to work with patients.
- 30. I desire more knowledge to improve my ability to work with patients.
- 40. I desire more experience to improve my ability to work with patients.

*Confidence – General (CG)*

- 2. I have all the experience I require to effectively interact with patients.
- 17. I have all the practical skills I require to effectively interact with patients.
- 22. I have learnt everything I need to know in order to effectively interact with patients.
- 32. I have all the theoretical knowledge I require to effectively interact with patients.

*Confidence – Communication (CC)*

- 6. I think I am good at creating a safe environment so that my patients feel comfortable enough to share information with me.
- 11. I feel confident sharing my formulations with patients.
- 21. I am good at providing clear messages to my patients.
- 34. I am good at listening to my patients with genuine curiosity.

*Uncertainty (Unc)*

- 8. Sometimes I am unsure if my planning for patients is the best possible way to proceed.
- 20. Sometimes I am unsure if I am interpreting my patients’ needs correctly.
- 27. Sometimes I am unsure how to handle the needs of patients.
- 31. Sometimes I am unsure that I properly understand the needs of patients.

*Stress interacting with patients (SiP)*

- 4. Sometimes after interacting with a patient I feel exhausted.
- 15. Sometimes I find interacting with patients to be stressful.
- 28. There are times when I feel distressed after communicating with a patient.
- 39. The pressure to meet the needs of my patients can sometimes feel overwhelming.

*Job satisfaction (JS)*

- 10. My work provides me with a lot of fulfilment.
- 18. My work means more to me than simply earning money.
- 25. I enjoy my work.
- 37. There are times when I find myself wishing that I did not have to go to work.

***Q37. Requires reverse scoring prior to averaging across items for this sub-scale***

**Section 2. Inter-correlations among items for RPQ sub-scales with four items**

Desire for improvement (DfI). Cronbach’s alpha = .81

|  | **Q5** | **Q19** | **Q30** | **Q40** |
| --- | --- | --- | --- | --- |
| **Q5** | 1 |  |  |  |
| **Q19** | .29* | 1 |  |  |
| **Q30** | .46* | .67* | 1 |  |
| **Q40** | .42* | .59* | .72* | 1 |

**p<.05*

Confidence – General (CG). Cronbach’s alpha = .83

|  | **Q2** | **Q17** | **Q22** | **Q32** |
| --- | --- | --- | --- | --- |
| **Q2** | 1 |  |  |  |
| **Q17** | .71* | 1 |  |  |
| **Q22** | .55* | .54* | 1 |  |
| **Q32** | .48* | .48* | .66* | 1 |

**p<.05*

Confidence – Communication (CC). Cronbach’s alpha = .75

|  | **Q6** | **Q11** | **Q21** | **Q34** |
| --- | --- | --- | --- | --- |
| **Q6** | 1 |  |  |  |
| **Q11** | .47* | 1 |  |  |
| **Q21** | .52* | .47* | 1 |  |
| **Q34** | .38* | .36* | .36* | 1 |

**p<.05*

Uncertainty (Unc). Cronbach’s alpha = .81

|  | **Q8** | **Q20** | **Q27** | **Q31** |
| --- | --- | --- | --- | --- |
| **Q8** | 1 |  |  |  |
| **Q20** | .45* | 1 |  |  |
| **Q27** | .36* | .47* | 1 |  |
| **Q31** | .47* | .69* | .58* | 1 |

**p<.05*

Stress interacting with patients (SiP). Cronbach’s alpha = .81

|  | **Q4** | **Q15** | **Q28** | **Q39** |
| --- | --- | --- | --- | --- |
| **Q4** | 1 |  |  |  |
| **Q15** | .58* | 1 |  |  |
| **Q28** | .50* | .67* | 1 |  |
| **Q39** | .46* | .42* | .54* | 1 |

**p<.05*

Job satisfaction. Cronbach’s alpha = .79

|  | **Q10** | **Q18** | **Q25** | **Q37** |
| --- | --- | --- | --- | --- |
| **Q10** | 1 |  |  |  |
| **Q18** | .44* | 1 |  |  |
| **Q25** | .77* | .48* | 1 |  |
| **Q37^1^** | .48* | .41* | .48* | 1 |

**p<.05 ^1^Note Q37 has been reverse scored*

**Section 3. Comparison among RPQ sub-scales for different groups.**

ANOVA for each sub-scale, with ‘samples’ (of medical students, mental health practitioners, and the general public) as the between participants factor.

| **Sub-scale** | **ANOVA result** |
| --- | --- |
| RC | *F*(2,383) = 34.66, *p* < .001 |
| DfI | *F*(2,383) = 77.93, *p* < .001 |
| CG | *F*(2,283) = 31.19, *p* < .001 |
| CC | *F*(2,283) = 1.13, *p* = .32 |
| Unc | *F*(2,283) = 33.25, *p* < .001 |
| SiP | *F*(4,91) = 4.01, *p* = .02 |
| JS | *F*(4,91) = 32.24, *p* < .001 |

Sub-scales: RC = Reflective capacity; DfI = Desire for improvement; CG = Confidence – general; CC = Confidence – communication; Unc = Uncertainty; SiC = Stress interacting with patients; JS = Job satisfaction

Follow up Bonferroni adjusted comparisons.

| **Sub-scale** | **Significant (*p* < .05) Adjusted comparisons** |
| --- | --- |
| RC | Med. students Vs. General public (p < .001), MHP Vs. General public (p < .001) |
| DfI | Med. students Vs. MHP (p = .002), Med students Vs. General public (p < .001), MHP Vs. General public (p < .001) |
| CG | Med. students Vs. General public (p < .001), MHP Vs. General public (p < .001) |
| CC | None. |
| Unc | Med. students Vs. MHP (p < .001), Med students Vs. General public (p < .001), MHP Vs. General public (p = .002) |
| SiP | Med. students Vs. MHP (p = .02) |
| JS | Med. students Vs. General public (p < .001), MHP Vs. General public (p < .001) |

Med. Students = Medical students; MHP = Mental health practitioners; General public = general public sample.

**Section 4. Cluster analysis dendrogram**

The dendrogram for the cluster analysis is shown below.

Five groups emerged as the best classification of the data.


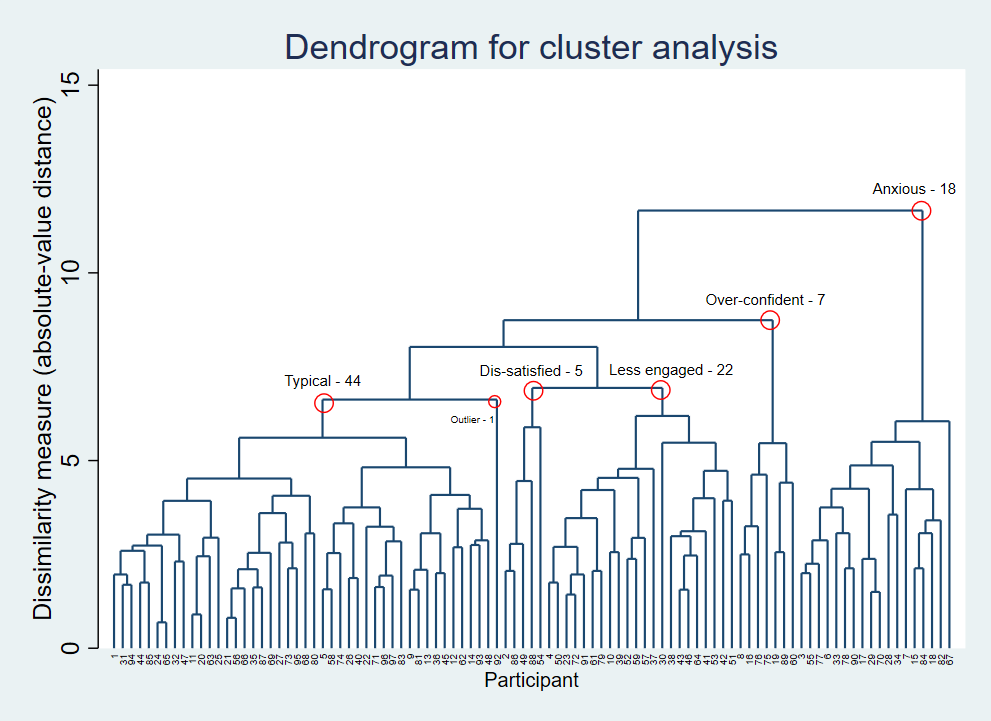


**Section 5. Statistical comparison of the cluster analysis groups.**

ANOVA for each sub-scale, with ‘cluster group’ as the between participants factor.

| **Sub-scale** | **ANOVA result** |
| --- | --- |
| RC | *F*(4,91) = 9.53, *p* < .001 |
| DfI | *F*(4,91) = 21.34, *p* < .001 |
| CG | *F*(4,91) = 27.55, *p* < .001 |
| CC | *F*(4,91) = 11.47, *p* < .001 |
| Unc | *F*(4,91) = 14.81, *p* < .001 |
| SiP | *F*(4,91) = 11.03, *p* < .001 |
| JS | *F*(4,91) = 19.79, *p* < .001 |

Sub-scales: RC = Reflective capacity; DfI = Desire for improvement; CG = Confidence – general; CC = Confidence – communication; Unc = Uncertainty; SiC = Stress interacting with patients; JS = Job satisfaction

Follow up Bonferroni adjusted comparisons.

| **Sub-scale** | **Significant (*p* < .05) Adjusted comparisons** |
| --- | --- |
| RC | Typical Vs. Less engaged (*p* = .005), Over-confident (*p* < .001); Over-confident Vs. Anxious (*p* < .001); Less engaged Vs Anxious (*p* = .002). |
| DfI | Typical Vs. Less engaged (*p* < .001), Over-confident (*p* < .001), Dis-satisfied (*p* = .02); Less engaged Vs. Anxious (*p* = .006); Over-confident Vs. Anxious (*p* < .001); Dis-satisfied Vs. Anxious (*p* = .006). |
| CG | Typical Vs. Less engaged (*p* < .001), Over-confident (*p* = .004), Anxious (*p* < .001); Less engaged Vs. Over-confident (*p* < .001), Dis-satisfied (*p* = .001), Anxious (*p* = .03); Over-confident Vs. Anxious (*p* < .001); Dis-satisfied Vs. Anxious (*p* < .001). |
| CC | Typical Vs. Less engaged (*p* < .001), Dis-satisfied (*p* = .012); Less engaged Vs. Over-confident (*p* < .001); Over-confident Vs. Dis-satisfied (*p* < .001), Anxious (*p* = .006). |
| Unc | Typical Vs. Over-confident (*p* < .001), Anxious (*p* < .001); Less engaged Vs. Over-confident (*p* =.007), Anxious (*p* < .001); Over-confident Vs. Anxious (*p* < .001); Dis-satisfied Vs. Anxious (*p* = .015). |
| SiP | Typical Vs. Over-confident (*p* = .032), Anxious (*p* < .001); Less engaged Vs. Over-confident (*p* = .002), Anxious (*p* = .064); Over-confident Vs. Dis-satisfied (*p* = .002), Anxious (*p* < .001). |
| JS | Typical Vs. Less engaged (*p* < .001), Dis-satisfied (*p* < .001), Anxious (*p* = .015); Less engaged Vs. Dis-satisfied (*p* < .001); Over-confident Vs. Dis-satisfied (*p* < .001); Dis-satisfied Vs. Anxious (*p* < .001). |

Sub-scales: RC = Reflective capacity; DfI = Desire for improvement; CG = Confidence – general; CC = Confidence – communication; Unc = Uncertainty; SiC = Stress interacting with patients; JS = Job satisfaction
